# A rare case of combined small cell lung carcinoma with heterologous component of rhabdomyosarcoma diagnosed by endobronchial ultrasound (EBUS)-guided biopsy

**DOI:** 10.1016/j.rmcr.2023.101899

**Published:** 2023-07-20

**Authors:** Diego Cedano, Jorge Cedano, Daffolyn Rachael Fels Elliott, Jasmeet Assi, Rashna Madan, Stephen Hyter, Lucas R. Pitts, Maykol R. Postigo Jasahui

**Affiliations:** a Division of Pulmonary, Critical Care, and Sleep Medicine, University of Kansas Medical Center, Kansas City, KS, USA; b Department of Pathology and Laboratory Medicine, University of Kansas Medical Center, Kansas City, KS, USA

**Keywords:** Combined small-Cell lung carcinoma, Rhabdomyosarcoma, Lung cancer

## Abstract

We describe an unusual case of combined small cell lung carcinoma (SCLC) with a heterologous sarcomatous component of rhabdomyosarcoma in a 61-year-old male smoker. The diagnosis was made using endobronchial ultrasound (EBUS)-guided fine needle aspiration and biopsy. This report highlights the challenges of diagnosing small round blue cell tumors in limited material and the importance of ancillary testing. The histologic diagnosis informed clinical management and therapy.

## 1 Introduction

Endobronchial ultrasound (EBUS)-guided biopsy is a valuable clinical tool to obtain lesional tissue for pathologic diagnosis and simultaneously perform nodal staging in lung cancer. Small biopsy samples pose a diagnostic challenge for small round blue cell tumors due to similar cytomorphology and a broad differential diagnosis [1]. The combination of small cell lung carcinoma (SCLC) with a heterologous sarcomatous component of rhabdomyosarcoma has rarely been described [2]. There is morphologic overlap between small cell carcinoma and rhabdomyosarcoma, and ancillary testing is necessary to make an accurate diagnosis and guide clinical management.

## 2 Case report

**Fig. 1:**
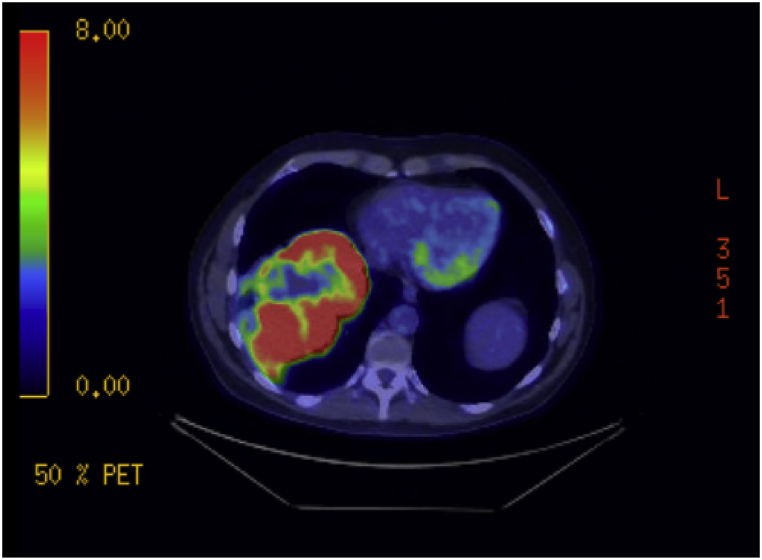
Chest positron emission tomography/computed tomography.

A 61-year-old man with a history of smoking (15 pack-years) presented with right upper quadrant pain and shortness of breath. Chest computerized tomography (CT) scan showed a large enhancing 15.4 cm mass in the right lung lower lobe, abutting the pericardium, lateral chest wall, and diaphragm, with surrounding ground-glass opacities and septal thickening. Positron emission tomography CT (PET/CT) scan demonstrated hypermetabolic uptake in the lung mass (SUV of 14.1) and mild uptake in a few mediastinal and right hilar lymph nodes (Fig. 1). Brain magnetic resonance imaging (MRI) exhibited no evidence of metastasis.

Hypermetabolic uptake (SUV of 14) in a large right lower lobe lung mass abutting the chest wall and pericardium.

EBUS-guided fine needle aspiration (FNA) was performed on the right lower lobe mass (7 passes) and lymph node stations 7, 4R and 11R (3–4 passes each). Cytology from the lung mass revealed a malignant small round blue cell tumor, but the neoplasm could not be further characterized due to limited material in the cell block. All lymph nodes were negative for malignancy. Repeat EBUS-guided FNA and transbronchial biopsies were performed to obtain additional material for ancillary testing.

**Fig. 2:**
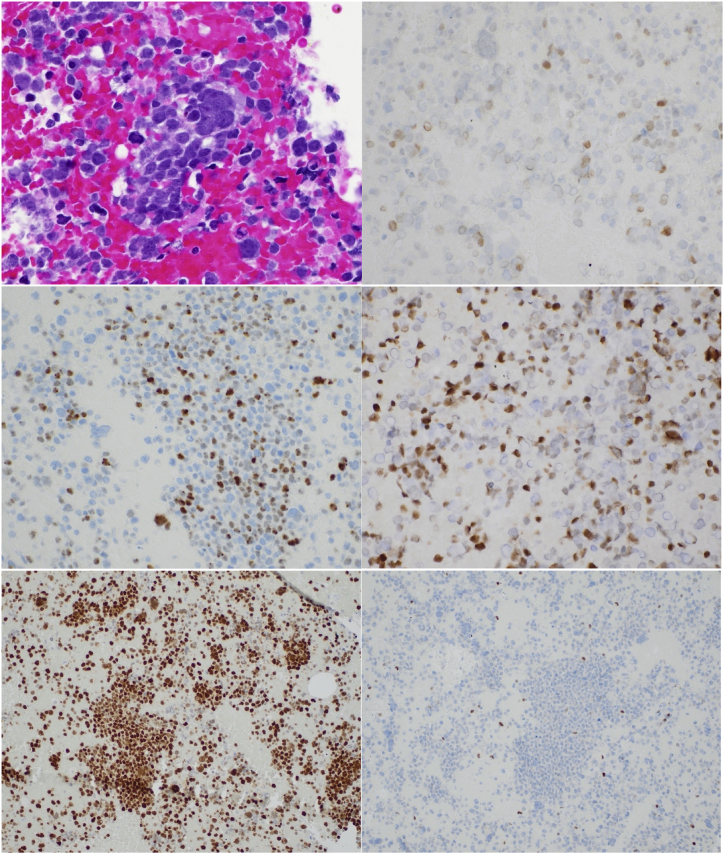
Histopathologic findings of combined small cell lung carcinoma with a heterologous component of rhabdomyosarcoma (A) Cell block shows a small round blue cell tumor with scant cytoplasm and nuclear pleomorphism (hematoxylin and eosin (H&E) stain, x 400). Immunohistochemistry shows a subset of tumor cells express (B) weak TTF-1 and (C) INSM-1, and a subset express (D) myogenin (x 400). All tumor cells show complete loss of nuclear (E) p53 and (F) retinoblastoma (Rb) protein (x 200). (For interpretation of the references to colour in this figure legend, the reader is referred to the Web version of this article.)

Microscopic sections and cytologic smear preparations showed a neoplasm composed of round cells with scant cytoplasm and nuclear pleomorphism with inconspicuous nucleoli (Fig. 2A). Immunohistochemical stains revealed that a subset of tumor cells expressed weak TTF-1 (Fig. 2B), focal cytokeratin CAM5.2, and were positive for neuroendocrine markers INSM-1 (Fig. 2C) and synaptophysin. Another population of tumor cells was strongly positive for myogenic markers desmin, myogenin (Fig. 2D) and MyoD1. All tumor cells showed complete loss of nuclear p53 and retinoblastoma (Rb) protein expression (Fig. 2E and F). SMARCA4/BRG1 and INI-1 demonstrated retained nuclear staining. Tumor cells were negative for pan-cytokeratin, p40, chromogranin, CD56, NUT1, SOX10 and CD45.

Fluorescence in situ hybridization (FISH) for *FOXO1* rearrangement was negative. Next-generation sequencing (NGS) using the Oncomine Comprehensive Assay v3 (Thermo Fisher) showed multiple copy number deletions in tumor suppressor genes, including *TP53* and *RB-1*, and a frameshift mutation in *EP300* A876Rfs*70. Copy number analysis also revealed striking amplification of *MYCL*.

**Fig. 3:**
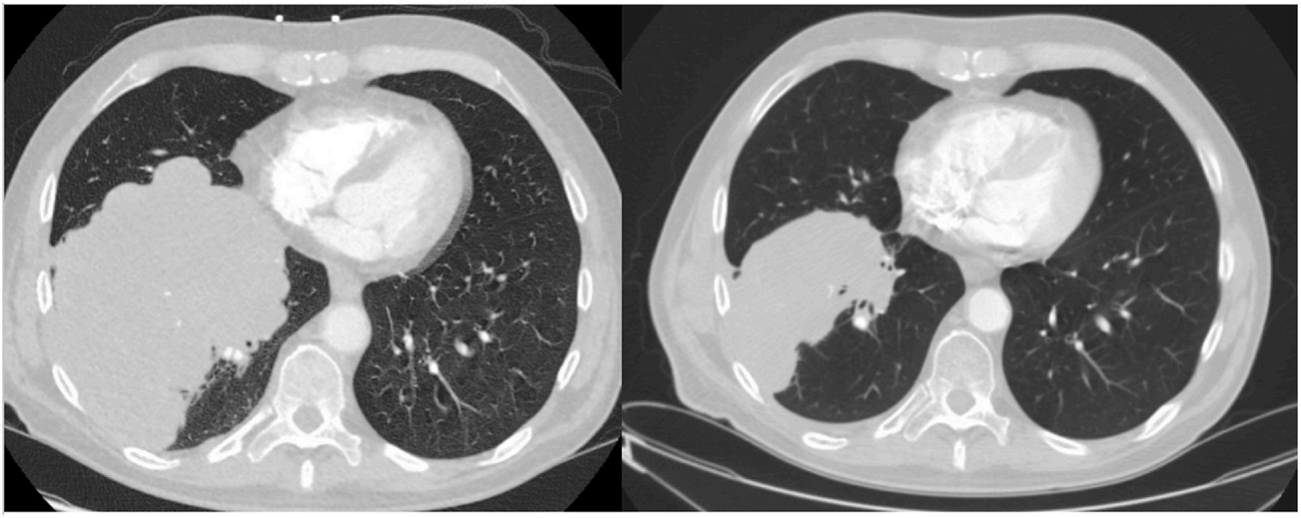
Chest computed tomography scan before and after four cycles of chemoradiation (A) Chest CT scan at diagnosis of combined SCLC revealed a 15.4 cm × 13.2 cm lobulated mass in the right lower lobe. (B) Repeat chest CT scan after four cycles of concurrent chemoradiation therapy showed decrease in size of the mass (10.5 cm × 6.3 cm).

The final pathologic diagnosis was high grade malignant neoplasm with neuroendocrine and rhabdomyosarcomatous differentiation, most consistent with combined SCLC with a heterologous sarcomatous component of rhabdomyosarcoma. The clinical disease stage was IIIA (cT4, cN0, cM0) by the American Joint Committee on Cancer (AJCC) TNM staging system and considered limited-stage disease by National Comprehensive Cancer Network (NCCN) SCLC guidelines [3]. Treatment was initiated with concurrent chemotherapy (cisplatin and etoposide) and radiation. Following four cycles of therapy, repeat chest CT scan showed partial interval decrease in size of the lung mass (Fig. 3).

## 3 Discussion

Combined small cell carcinoma with heterologous sarcomatous elements has rarely been described in the lung [2,4], and this report highlights the challenges of making an accurate diagnosis in small biopsy material. Combined SCLC is defined in the 2021 WHO Classification of Thoracic Tumors as SCLC with an additional component of non-small cell carcinoma and/or rarely heterologous sarcomatous element(s) [5]. The differential diagnosis includes carcinosarcoma, which is defined as non-small cell carcinoma with associated heterologous sarcomatous component(s) [5]. Although we cannot exclude an unsampled component of non-small cell carcinoma in this large 15.4 cm lung mass, the presence of small cell carcinoma differentiates combined SCLC from carcinosarcoma. A component of small cell carcinoma is important to recognize because of the differences in clinical management guidelines and chemotherapy regimens [3].

The histologic differential diagnosis also includes primary pulmonary rhabdomyosarcoma, which is predominantly a pediatric malignancy and rarely reported in adults [[6], [7], [8]]. Adult primary pulmonary rhabdomyosarcoma may arise in the setting of hereditary syndromes such as neurofibromatosis [9]. Alveolar and embryonal subtypes of rhabdomyosarcoma are composed of primitive round cells and have morphologic overlap with small cell carcinoma. Ancillary testing with a panel of immunostains and molecular testing is necessary for diagnosis. By immunohistochemistry, rhabdomyosarcoma expresses myogenic markers including desmin, myogenin and MyoD1. The majority of alveolar rhabdomyosarcomas demonstrate fusions involving the *FOXO1* gene [10], which was not detected in our case.

Although cytology is sufficient to diagnose SCLC in most clinical scenarios, making a diagnosis of combined SCLC is very challenging and requires ancillary testing [1]. Small round blue cell tumors have a broad differential diagnosis that includes small cell carcinoma, lymphoma, melanoma, Merkel cell carcinoma, sarcoma, and others. In our case, there was insufficient cellularity in the cell block following the first EBUS-guided FNA procedure, and additional needle passes were required to obtain sufficient material for immunohistochemistry and molecular testing. Transbronchial biopsies were also obtained during the second EBUS procedure, but histologic evaluation was limited by crush artifact which is common in SCLC. By immunohistochemistry, the expression of neuroendocrine markers (INSM-1 and synaptophysin) and aberrant expression of p53 and Rb supported a component of small cell carcinoma, while the expression of skeletal muscle markers (myogenin and MyoD1) supported a component of rhabdomyosarcoma.

NGS provided additional information to support the histologic diagnosis of small cell carcinoma in this case. Copy number analysis revealed losses in both *TP53* and *RB1*, which validated the immunohistochemistry findings of complete loss of p53 and Rb nuclear expression. Biallelic loss of function mutations in the tumor suppressors *TP53* and *RB1* drive oncogenesis in a majority (>90%) of SCLC [11]. Detection of concurrent *TP53* and *RB1* alterations is helpful to support a diagnosis of SCLC in challenging cases with partial or absent expression of neuroendocrine markers. In addition, a frameshift mutation was detected in *EP300,* a *NOTCH* family gene that encodes p300 and is involved in transcriptional coactivation of the Notch signaling pathway. Inactivating mutations in *NOTCH* family genes have been described in ∼25% of SCLC and appear to be mutually exclusive [11,12]. Finally, frequent copy number alterations reported in SCLC include mutually exclusive amplifications in *MYC* family genes such as *MYCL* [12], which showed marked amplification in our case.

Due to rapid tumor proliferation, SCLC tends to present clinically as a large mass with early lymph node involvement and spread to distant metastatic sites. Chest imaging typically shows a large hilar mass and bulky mediastinal lymphadenopathy, but patients can present with a broad spectrum of radiologic findings like in other lung cancers. Our patient presented with a large lung mass without nodal involvement, which reflects the observation that combined SCLC may be more likely to present with limited stage disease than conventional SCLC [3,13].

Given its rarity, there are no specific management guidelines for combined SCLC with a heterologous component of rhabdomyosarcoma, but the NCCN Clinical Practice Guidelines recommend therapy for combined SCLC be based on the component of SCLC [3]. The treatment strategy in this case was to primarily target the SCLC component using concurrent chemoradiation with cisplatin and etoposide. Following four cycles of therapy, despite a partial response, a fairly large residual tumor mass persisted on chest imaging. The patient was not deemed a surgical candidate following review at multidisciplinary tumor board. Considerations for further management after the completion of the initial chemoradiation include additional radiation therapy and immunotherapy. Repeat biopsy using an alternative sampling modality such as CT-guided core biopsy could be helpful to re-evaluate the residual tumor composition.

## 4 Conclusion

Combined SCLC with a heterologous component of rhabdomyosarcoma is a rare diagnosis that requires ancillary testing. Sufficient tissue must be obtained by the bronchoscopist, and helpful techniques can include additional FNA passes to generate a cell block and targeted transbronchial biopsies with mini-forceps and/or cryo-biopsies. Accurate pathologic diagnosis is critical to guide clinical management of combined SCLC, which is primarily driven by the small cell carcinoma component but remains challenging.

## Funding

This research did not receive any specific grant from founding agencies in the public, commercial, or non-for-profit sector.

## Declaration of competing interest

The authors have no potential conflicts of interest to disclose.
